# Supplementary material for: Exploring the links between social connection and physical functioning among older Adults: A network analysis
Source: PLoS One. 2026 Mar 23;21(3):e0342656. doi: 10.1371/journal.pone.0342656 (PMC13008092; doi:10.1371/journal.pone.0342656)
Supplement: S1 Table — (ZIP) [file pone.0342656.s001.zip › S1 Fig.pdf]

S1 Fig. Diagram of Analytic Sample. Health and Retirement Study (2014/2016)

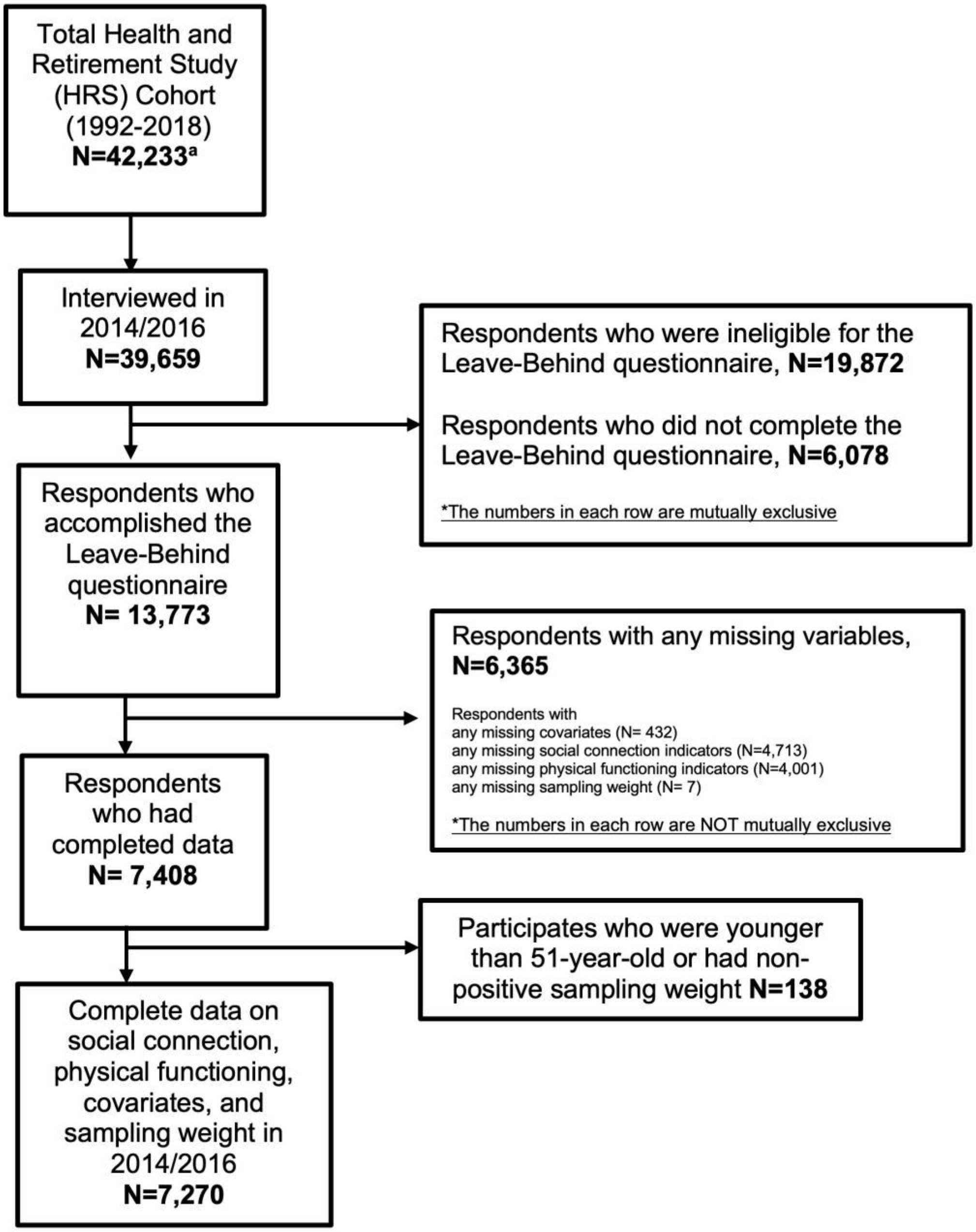

Note. [a] Includes decedent participants.
