# Supplementary material for: Exploring the links between social connection and physical functioning among older Adults: A network analysis
Source: PLoS One. 2026 Mar 23;21(3):e0342656. doi: 10.1371/journal.pone.0342656 (PMC13008092; doi:10.1371/journal.pone.0342656)
Supplement: S1 Table — (ZIP) [file pone.0342656.s001.zip › S1 Table.pdf]

S1 Table. Demographic Characteristics of the Analytic Sample Compared to the Excluded Respondents[1].

Characteristics	Analytic sample (N=7,270)	Excluded sample (N=6,503)	Test score (P-value)
**Age, Mean (SE)***	67.30 (0.24)	68.0 (0.29)	3.52 (0.0004)
**Sex, N (weight%)***			51.17 (<0.0001)
Female	4,160 (51.83)	4,110 (56.52)	
**Race/Ethnicity, N (weight%)**			273.25 (<.0001)
Hispanic	710 (7.47)	992 (12.20)	
Non-Hispanic White	5,265 (81.20)	3,850 (68.32)	
Non-Hispanic Black	1,032 (7.48)	1,371 (14.32)	
Others	263 (3.86)	288 (5.16)	
Missing	0	2	
**Education, N (weighted %)**			389.13 (<.0001)
Less than high school	734 (7.64)	1,332 (18.75)	
High school degree or GED	2,394 (30.22)	2,281 (34.43)	
Some college	1,989 (28.28)	1,586 (24.07)	
College or more	2,153 (33.85)	1,302 (22.75)	
Missing	0	2	
**Marital status, N (weighted %)**			35.82 (<.0001)
Partnered	4,745 (68.29)	3,918 (59.59)	
Not partnered	2,525 (31.71)	2,576 (40.41)	
Missing	0	9	
***Household income (in USD), N (weighted%)**			432.91 (<.0001)
≤ $22,760	1,453 (15.72)	2,230 (31.98)	
$22,761- $40,992	1,456 (16.05)	1,394 (20.04)	
$40,993- $66,130	1,453 (18.98)	1,074 (15.67)	
$66,131-$111,612	1,454 (22.31)	933 (15.81)	
> US $111,612	1,454 (26.94)	899 (16.49)	
***Total wealth (in USD), N (weighted%)**			317.01 (<.0001)
≤ $28,950	1,454 (17.68)	2,006 (28.89)	
$ 28,951-$135,250	1,454 (19.28)	1,468 (20.64)	
$135,251-$323,200	1,455 (19.50)	1,202 (17.62)	
$323,201-$752,200	1,453 (20.94)	957 (16.82)	
> $752,200	1,454 (22.59)	870 (16.03)	

Note. [1] Excluded respondents were those who completed 2014/2016 HRS PLBQ, but excluded from the analytic sample because they had missingness and non-positive sampling weight. [2] variables with * did not have any missing value.
