# Supplementary material for: Exploring the links between social connection and physical functioning among older Adults: A network analysis
Source: PLoS One. 2026 Mar 23;21(3):e0342656. doi: 10.1371/journal.pone.0342656 (PMC13008092; doi:10.1371/journal.pone.0342656)
Supplement: S1 Table — (ZIP) [file pone.0342656.s001.zip › S2 Fig .pdf]

S2 Fig Pearson Correlation Matrix of 34 Nodes: 2014/2016 Health and Retirement Study (n=7,270).

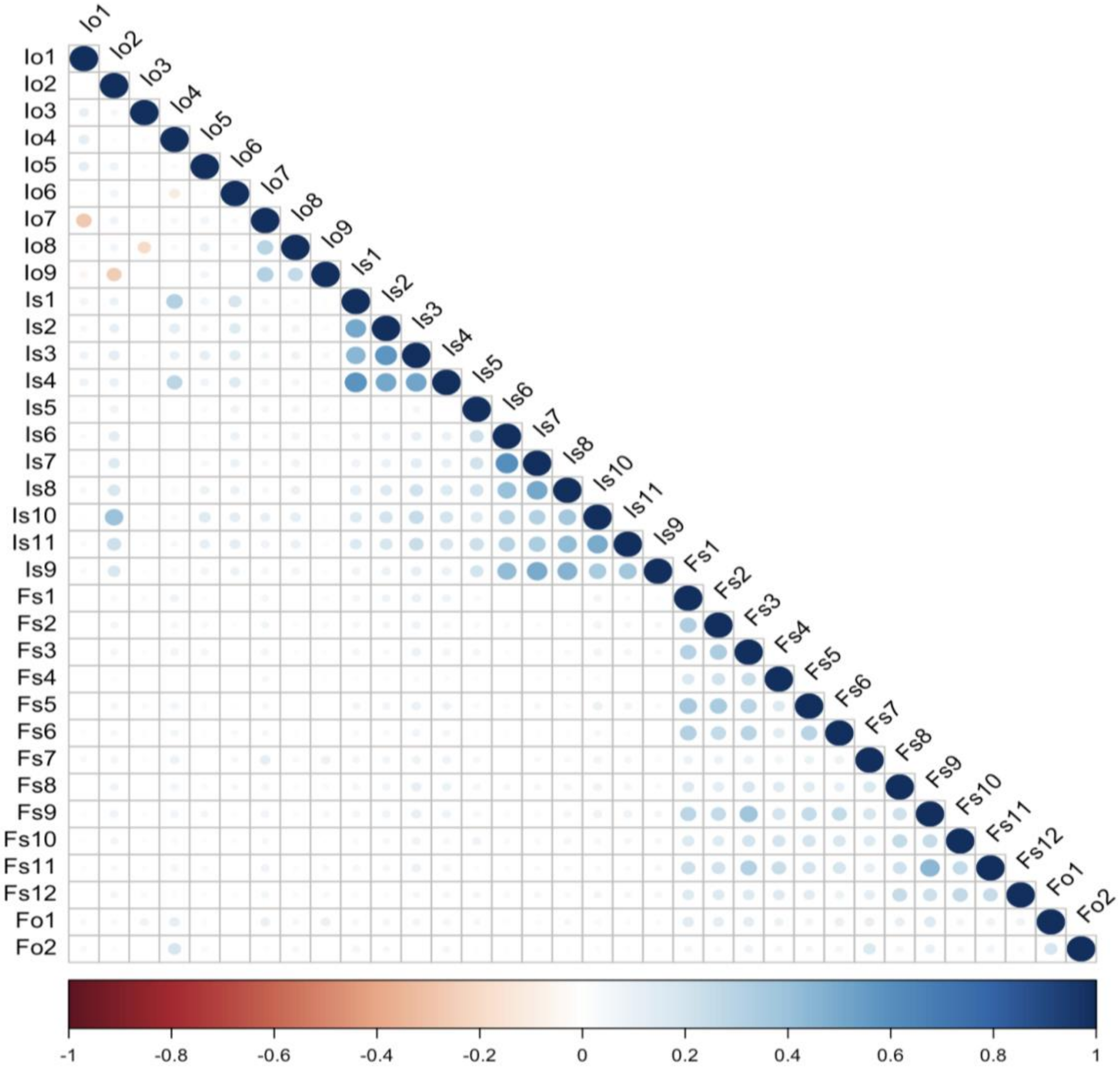

Note: The color gradient goes from blue (positive correlations) to red (negative correlations). Stronger correlation is represented by darker shades.
