# Supplementary material for: Exploring the links between social connection and physical functioning among older Adults: A network analysis
Source: PLoS One. 2026 Mar 23;21(3):e0342656. doi: 10.1371/journal.pone.0342656 (PMC13008092; doi:10.1371/journal.pone.0342656)
Supplement: S1 Table — (ZIP) [file pone.0342656.s001.zip › S2 Table.pdf]

S2 Table. Item Wording, Response Categories, and Recoding of the Nodes in the Network: Health and Retirement Study, 2014/2016

Node name	Description in figure legends	Original variable in the HRS and question wording	Original response options	Re-coded Response
**Construct of social connection: objective measures**				
**Io1**	**No children**	**LB 006** Do you have any living children?	1=Yes<br>0=No	0=Yes<br>1=No
**Io2**	**No friends**	**LB 014** Do you have any friends?	1=Yes<br>0=No	0=Yes<br>1=No
**Io3**	**No other family members**	**LB 010** Do you have any other immediate family, for example, any brothers or sisters, parents, cousins or grandchildren?	1=Yes<br>0=No	0=Yes<br>1=No
**Io4**	**Not partnered**	**MSTAT** What's your marital status?	1= Married or partnered,<br>2= Married, spouse absent,<br>3=Partnered,<br>4= Separated,<br>5=Divorced,<br>6=Separated/divorced,<br>7=Widowed,<br>8=Never married	0= Married or partnered<br>1= Other status
**Io5**	**Few activities**	At least doing one type of social activity several times a month.<br>**LB001B** do activities with younger generation;<br>**LB001C** volunteer with children;<br>**LB001D** volunteer/charity work;<br>**LB001E** attend courses;<br>**LB001F** go to sport, social, other club;<br>**LB001G** attend meeting of non-religious interest group;<br>**LB001U** participate community art group;	7 points Likert scale<br><br>1=Daily,<br>2=Several times a week,<br>3=Once a week,<br>4=Several times a month,<br>5=At least once a month,<br>6=Not in the last month,<br>7=Never/Not Relevant	0=Having more than 1 social activity that the participants attend several times a month<br><br>1=Not having more than 1 social activity that the participants attend several times a month

		**B082** participate in religious event		
**Io6**	**Not close partner**	**LB 005** How close is your relationship with your spouse or partner **?**	<u>4 points Likert Scale</u><br><br>1=Very close<br>2=Quite close<br>3=Not very close<br>4=Not at all close	0=Very close or quite close<br>1= Not very close or not at all close<br><br>If R didn't have a spouse/partner, we imputed 0 from them
**Io7**	**Low con child**	On average, how often do you do each of the following?<br>LB 008A Meet up (include both arranged and chance meetings)<br>LB 008B Speak on the phone<br>LB 008C Write or email<br>LB 008D Communicate by Skype, Facebook, or other social media	<u>6 points Likert Scale</u><br>1=Three or more times a week,<br>2=Once or twice a week,<br>3=Once or twice a month, 4=Every few months,<br>5=Once or twice a year,<br>6=Less than once a year or never	A summation score of 4 types of contact was created.<br><br>0= sum of contact frequency not lower than the median frequency of the full sample<br>1=sum of contact frequency lower than the median frequency of the full sample<br><br>If R didn't have children, we imputed 0 for them
**Io8**	**Low con fam**	On average, how often do you do each of the following?<br>**LB 012A** Meet up (include both arranged and chance meetings)<br>**LB 012B** Speak on the phone<br>**LB 012C** Write or email<br>**LB 012D** Communicate by Skype, Facebook, or other social media	<u>6 points Likert Scale</u><br>1=Three or more times a week,<br>2=Once or twice a week,<br>3=Once or twice a month, 4=Every few months,<br>5=Once or twice a year,<br>6=Less than once a year or never	A summation score of 4 types of contact was created.<br><br>0= sum of contact frequency not lower than the median frequency of the full sample<br>1=sum of contact frequency lower than the median frequency of the full sample<br><br>If R didn't have other family member,, we imputed 0 for them

**Io9**	**Low con fri**	On average, how often do you do each of the following?<br>**LB 016A** Meet up (include both arranged and chance meetings)<br>**LB 016B** Speak on the phone<br>**LB 016C** Write or email<br>**LB 016D** Communicate by Skype, Facebook, or other social media	<u>6 points Likert Scale</u><br>1=Three or more times a week,<br>2=Once or twice a week,<br>3=Once or twice a month, 4=Every few months,<br>5=Once or twice a year,<br>6=Less than once a year or never	A summation score of 4 types of contact was created.<br><br>0= sum of contact frequency not lower than the median frequency of the full sample<br>1=sum of contact frequency lower than the median frequency of the full sample<br><br>If R didn't have friend, we imputed 0 for them
**Construct of social connection: subjective measures**				
**Is1**	**Lack companionship**	**LB 019a** How much of the time do you feel you lack companionship?	<u>3 points Likert Scale</u><br>1 = hardly ever or never<br>2 = some of the time<br>3 = often	0=Hardly ever or never have the feeling<br>1=Often or some of the time have the feeling
**Is2**	**Left out**	**LB 019b** How much of the time do you feel left out?	<u>3 points Likert Scale</u><br>1 = hardly ever or never<br>2 = some of the time<br>3 = often	0=Hardly ever or never have the feeling<br>1=Often or some of the time have the feeling
**Is3**	**Isolated**	**LB 019c** How much of the time do you feel isolated from others?	<u>3 points Likert Scale</u><br>1 = hardly ever or never<br>2 = some of the time<br>3 = often	0=Hardly ever or never have the feeling<br>1=Often or some of the time have the feeling
**Is4**	**Alone**	**LB 019e** How much of the time do you feel alone?	<u>3 points Likert Scale</u><br>1 = hardly ever or never<br>2 = some of the time<br>3 = often	0=Hardly ever or never have the feeling<br>1=Often or some of the time have the feeling
**Is5**	**Not in tune**	**LB 019d** How much of the time do you feel that you are "in tune" with the people around you?	<u>3 points Likert Scale</u><br>1 = hardly ever or never<br>2 = some of the time<br>3 = often	1)Reverse coded first<br><br>2)0=Hardly ever or never have the feeling<br>1=Often or some of the time have the feeling
**Is6**	**No ppl talk**	**LB 019f** How much of the time do you feel that there are people you can talk to?	<u>3 points Likert Scale</u><br>1 = hardly ever or never<br>2 = some of the time<br>3 = often	1)Reverse coded first<br><br>2)0=Hardly ever or never have the feeling

				1=Often or some of the time have the feeling
**Is7**	**No ppl turn**	**LB 019g** How much of the time do you feel that there are people you can turn to?	3 points Likert Scale<br>1 = hardly ever or never<br>2 = some of the time<br>3 = often	1)Reverse coded first<br><br>2)0=Hardly ever or never have the feeling<br>1=Often or some of the time have the feeling
**Is8**	**Not understood**	**LB 019h** How much of the time do you feel that there are people who really understand you?	3 points Likert Scale<br>1 = hardly ever or never<br>2 = some of the time<br>3 = often	1)Reverse coded first<br><br>2)0=Hardly ever or never have the feeling<br>1=Often or some of the time have the feeling
**Is9**	**Not ppl close**	**LB 019i** How much of the time do you feel that there are people you feel close to?	3 points Likert Scale<br>1 = hardly ever or never<br>2 = some of the time<br>3 = often	1)Reverse coded first<br><br>2)0=Hardly ever or never have the feeling<br>1=Often or some of the time have the feeling
**Is10**	**Not part group**	**LB 019j** How much of the time do you feel part of a group of friends?	3 points Likert Scale<br>1 = hardly ever or never<br>2 = some of the time<br>3 = often	1)Reverse coded first<br><br>2)0=Hardly ever or never have the feeling<br>1=Often or some of the time have the feeling
**Is11**	**Can't found common**	**LB 019k** How much of the time do you feel that you have a lot in common with the people around you?	3 points Likert Scale<br>1 = hardly ever or never<br>2 = some of the time<br>3 = often	1)Reverse coded first<br><br>2)0=Hardly ever or never have the feeling<br>1=Often or some of the time have the feeling
**Construct of physical functioning: objective measures**				
**Fo1**	**Incomplete balance tests**	**RwBALSEMI** standing time for the semi- tandem balance test<br>**RwBALFUL** standing time for the full tandem balance test	A respondent failed the semi tandem balance test for standing less than 10s.<br><br>A respondent failed the full tandem balance test for standing less than 30s (for 65yr+) or 60s (for < 65yr)	A summed score of standing time was created<br><br>Range:<br>Cutoff line: 40s for 65yr+<br><br>0=No , the observation finished the 2 balance tests

				1=Yes, the observation failed to finished the 2 balance tests
**Fo2**	**Low grip strength**	**RwGRPR (max grip strength of right hand)**<br>**RwGRPL (max grip strength of left hand)**<br>**RwDOM (dominant hand)**<br><br>respondents who did not perform grip strength tests due to surgery, injury, or medical reasons	We used the maximum grip strength reported for the dominant hand (either left or right) or maximum of left- and right-handed grip strength when both hands were equally dominant.	Range:<br>Median: 29.0kg<br>Pearson correlation of left and right grip strength = 0.76<br><br>0=No , the grip strength was greater than or equal to the median of the sample<br>1=Yes, the grip strength was lower than the median of the sample
**Construct of physcial functioning: subjective measures**				
**Fs1**	**Walk**	**RwWALKRA** Do you have any difficulty in walking?	0= No<br>1=Yes	0=No, the respondent did not have any difficulty 1=Yes, the respondent has some difficulty or can't do
**Fs2**	**Dress**	**RwDRESSA** Do you have any difficulty in dressing?	0= No<br>1=Yes	0=No, the respondent did not have any difficulty 1=Yes, the respondent has some difficulty or can't do
**Fs3**	**Bath**	**RwBATHA** Do you have any difficulty in bathing?	0= No<br>1=Yes	0=No, the respondent did not have any difficulty<br>1=Yes, the respondent has some difficulty or can't do
**Fs4**	**Eat**	**RwEATA** Do you have any difficulty in eating?	0= No<br>1=Yes	0=No, the respondent did not have any difficulty<br>1=Yes, the respondent has some difficulty or can't do
**Fs5**	**Bed**	**RwBEDA** Do you have any difficulty in getting in and out of bed?	0= No<br>1=Yes	0=No, the respondent did not have any difficulty<br>1=Yes, the respondent has some difficulty or can't do
**Fs6**	**Toilet**	**RwTOILTA** Do you have any difficulty in toilet?	0= No<br>1=Yes	0=No, the respondent did not have any difficulty 1=Yes, the

				respondent has some difficulty or can't do
**Fs7**	**Map**	**RwMAPA** Do you have any difficulty in using a map?	0= No<br>1=Yes	0=No, the respondent did not have any difficulty 1=Yes, the respondent has some difficulty or can't do
**Fs8**	**Money**	**RwMONEYA** Do you have any difficulty in managing money?	0= No<br>1=Yes	0=No, the respondent did not have any difficulty 1=Yes, the respondent has some difficulty or can't do
**Fs9**	**Shop**	**RwSHOPA** Do you have any difficulty in shopping for groceries?	0= No<br>1=Yes	0=No, the respondent did not have any difficulty 1=Yes, the respondent has some difficulty or can't do
**Fs10**	**Medication**	**RwMEDSA** Do you have any difficulty in taking medications?	0= No<br>1=Yes	0=No, the respondent did not have any difficulty 1=Yes, the respondent has some difficulty or can't do
**Fs11**	**Meal**	**RwMEALSA** Do you have any difficulty in preparing hot meals?	0= No<br>1=Yes	0=No, the respondent did not have any difficulty 1=Yes, the respondent has some difficulty or can't do
**Fs12**	**Cellphone**	**RwPHONE** Do you have any difficulty in using a telephone?	0= No<br>1=Yes	0=No, the respondent did not have any difficulty 1=Yes, the respondent has some difficulty or can't do
