# Supplementary material for: Exploring the links between social connection and physical functioning among older Adults: A network analysis
Source: PLoS One. 2026 Mar 23;21(3):e0342656. doi: 10.1371/journal.pone.0342656 (PMC13008092; doi:10.1371/journal.pone.0342656)
Supplement: S1 Table — (ZIP) [file pone.0342656.s001.zip › S3 Fig .pdf]

S3 Fig Plot of assortativity index for the ablated networks

S3-a Fig Assortativity for physical functioning/ social connectionof the ablated network

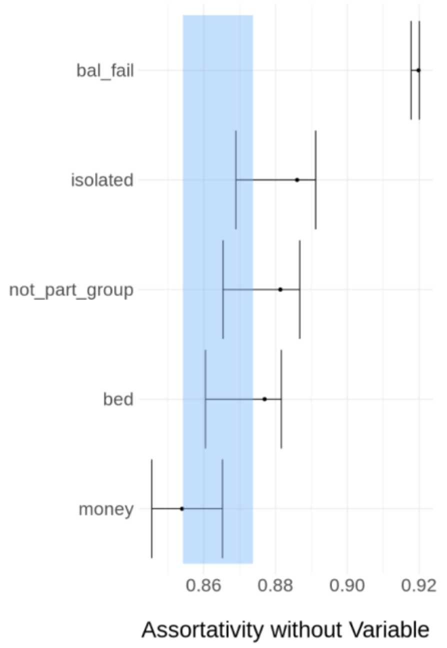

S3-b Fig Assortativity for objective/subjective of the ablated network

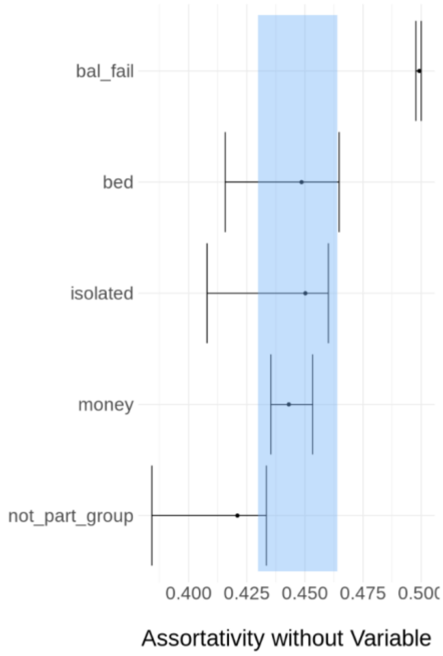

Note. On the X-Axis is the assortativity of the ablated network. On the Y-Axis are the variables that were removed from the network for each respective ablation test. The blue band is the 95% bootstrap confidence interval of full network assortativity, across 10,000 bootstrap samples. **S3-a Fig** is the assortativity for physical functioning/social connection of the ablated network. **S3-b Fig** is the assortativity of objective/subjective in the ablated network.
