# Supplementary material for: Exploring the links between social connection and physical functioning among older Adults: A network analysis
Source: PLoS One. 2026 Mar 23;21(3):e0342656. doi: 10.1371/journal.pone.0342656 (PMC13008092; doi:10.1371/journal.pone.0342656)
Supplement: S1 Table — (ZIP) [file pone.0342656.s001.zip › S3 Table.pdf]

S3 Table. Details of Software Packages Used in the Study

**Statistical Analysis**	**Purpose of the analysis**	**Package Used**
Data management	Indicator operationalization, dichotomizing all indicators	*DATA procedure* in SAS
Descriptive analysis	Calculating the mean (weighted standard deviation), and frequency (weighted percentage) of covariates	*PROC MEANS* and *PROC FREQ procedure* in SAS
Indicator exploration	Estimating the mean, standard deviation of each indicators	*PROC MEANS procedure* in SAS
Correlation exploration	Estimating the correlation between each pair of indicators	R package *corrplot*
Statistical regularization	Generating the edgelist of the network	R package *glmnet*
Assortativity	Calculating the assortativity coefficient of a given network	The *assortativity* function in R package *igraph*
Community detection	Identifying communities in the network	The *cluster_spinglass* function in R package *igraph*
Centrality	Calculating the betweenness centrality and strength of all nodes in a given network	The *strength* and *betweenness* functions in R package *igraph*
Null model	Applying configuration model to generate reference line for each parameter	The *degree_sequence_game function* in R package *igraph*
Network visualization	Applying the Fructerman-Reingold (default) algorithm for layout of the network	R package *igraph*
