# Supplementary material for: Exploring the links between social connection and physical functioning among older Adults: A network analysis
Source: PLoS One. 2026 Mar 23;21(3):e0342656. doi: 10.1371/journal.pone.0342656 (PMC13008092; doi:10.1371/journal.pone.0342656)
Supplement: S1 Table — (ZIP) [file pone.0342656.s001.zip › S4 Fig.pdf]

S4 Fig. Sensitivity Analysis of Community Alignment

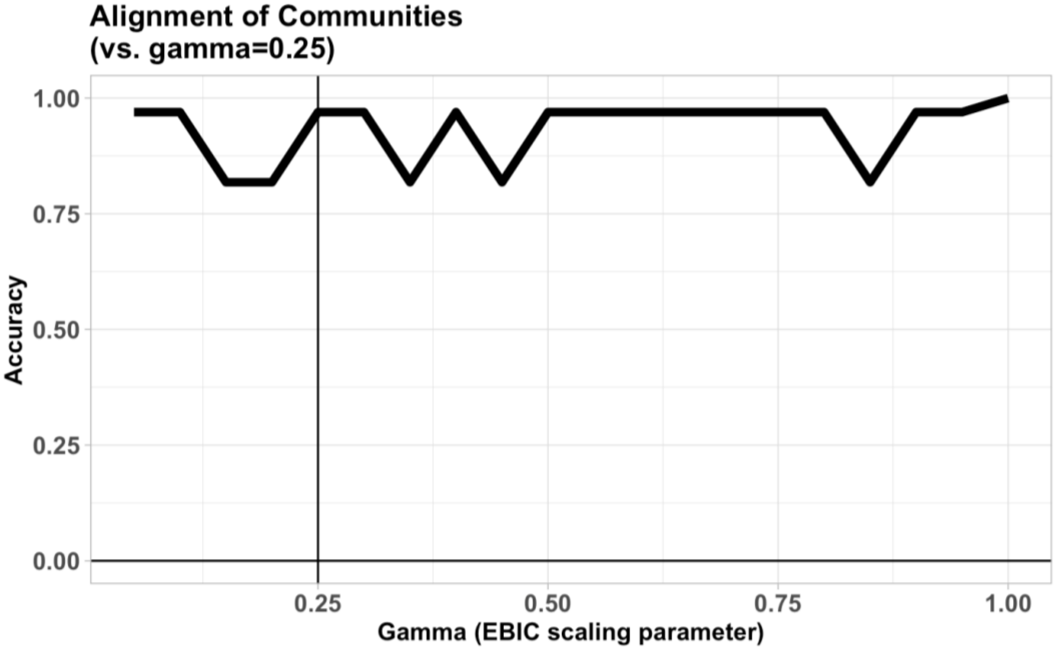

Note. On the X-Axis are different values of gamma from 0.05 to 1.00 with the interval of 0.05. The vertical black line is the value of gamma (0.25) used in the present study. On the Y-axis are the values of the accuracy of communities detected in networks with different values of gamma compared to communities detected with gamma=0.25.
