# Supplementary material for: Exploring the links between social connection and physical functioning among older Adults: A network analysis
Source: PLoS One. 2026 Mar 23;21(3):e0342656. doi: 10.1371/journal.pone.0342656 (PMC13008092; doi:10.1371/journal.pone.0342656)
Supplement: S1 Table — (ZIP) [file pone.0342656.s001.zip › S4 Table.pdf]

S4 Table. Descriptive Statistical Analysis of All Indicators in the Network

Node name	legend	Mean	SD	Min	Max
**Objective social connection**					
Io1	No children	0.13	0.34	0	1
Io2	No friends	0.09	0.28	0	1
Io3	No other family members	0.07	0.25	0	1
Io4	Not partnered	0.35	0.48	0	1
Io5	Few activities	0.23	0.42	0	1
Io6	Not close partner	0.07	0.25	0	1
Io7	Low contact with child	0.37	0.48	0	1
Io8	Low contact with family	0.42	0.49	0	1
Io9	Low contact with friends	0.47	0.50	0	1
**Subjective social connection**					
Is1	Lack companionship	0.43	0.49	0	1
Is2	Left out	0.40	0.49	0	1
Is3	Isolated	0.31	0.46	0	1
Is4	Alone	0.41	0.49	0	1
Is5	Not in tune	0.10	0.30	0	1
Is6	No ppl talk	0.06	0.24	0	1
Is7	No ppl turn	0.06	0.23	0	1
Is8	Not understood	0.07	0.26	0	1
Is9	Not ppl close	0.05	0.22	0	1
Is10	Not part group	0.16	0.36	0	1
Is11	Not in common with others	0.16	0.30	0	1
**Objective physical functioning**					
Fo1	Incomplete balance tests	0.30	0.46	0	1
Fo2	Low grip strength	0.48	0.50	0	1
**Subjective physical functioning**					
Fs1	Walk	0.03	0.17	0	1
Fs2	Dress	0.06	0.24	0	1
Fs3	Bath	0.03	0.17	0	1
Fs4	Eat	0.01	0.10	0	1
Fs5	Bed	0.03	0.17	0	1
Fs6	Toilet	0.03	0.18	0	1
Fs7	Map	0.10	0.30	0	1
Fs8	Money	0.03	0.18	0	1
Fs9	Shop	0.05	0.21	0	1
Fs10	Medication	0.02	0.13	0	1
Fs11	Meal	0.02	0.15	0	1
Fs12	Cellphone	0.02	0.13	0	1
