# Supplementary material for: Exploring the links between social connection and physical functioning among older Adults: A network analysis
Source: PLoS One. 2026 Mar 23;21(3):e0342656. doi: 10.1371/journal.pone.0342656 (PMC13008092; doi:10.1371/journal.pone.0342656)
Supplement: S1 Table — (ZIP) [file pone.0342656.s001.zip › S5 Fig.pdf]

S5 Fig Sensitivity Analysis of Assortativity of Network

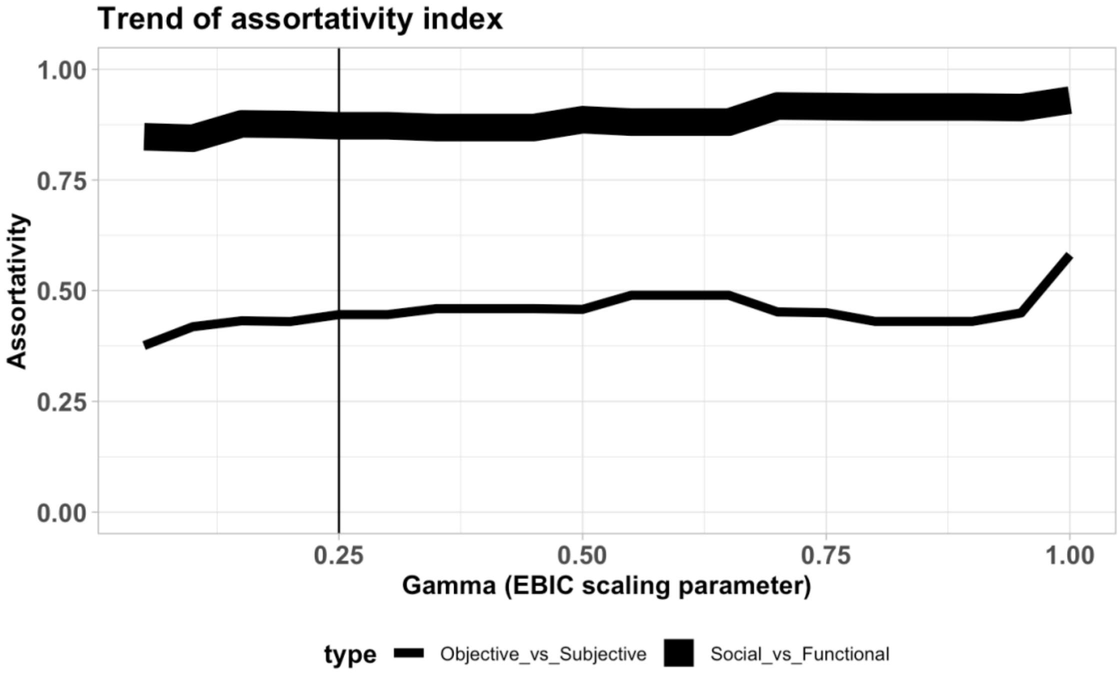

Note. On the X-Axis are different values of gamma from 0.05 to 1.00 with the interval of 0.05. The vertical black line is the value of gamma used in the present study. On the Y-axis are the values of the assortativity between different values of gamma. The thinner line is the trend of assortativity of different measurements, while the thicker line is the assortativity of different constructs.
