# Supplementary material for: Exploring the links between social connection and physical functioning among older Adults: A network analysis
Source: PLoS One. 2026 Mar 23;21(3):e0342656. doi: 10.1371/journal.pone.0342656 (PMC13008092; doi:10.1371/journal.pone.0342656)
Supplement: S1 Table — (ZIP) [file pone.0342656.s001.zip › S5 Table.pdf]

S5 Tables of 95% Confidence Interval of Betweenness Centrality and Strength

S5-1 Table. 95% Confidence Interval of Betweenness Centrality from 10,000 Bootstrap Results

Node	Observed Network		Null Network (average)		p (observed > null)
	Bootstrap Mean	Bootstrap 95% CI	Bootstrap Mean	Bootstrap 95% CI	
isolated	0.268	[0.24,0.271]	0.102	[0.093,0.11]	0.0034
not_part_group	0.22	[0.21,0.233]	0.086	[0.077,0.1]	0.0034
bal_fail	0.156	[0.155,0.173]	0.05	[0.04,0.062]	0.0034
bed	0.109	[0.104,0.116]	0.045	[0.039,0.051]	0.0034
money	0.097	[0.094,0.107]	0.049	[0.04,0.062]	0.0034
medication	0.071	[0.056,0.072]	0.057	[0.05,0.064]	0.0034
low_con_fam	0.051	[0.043,0.051]	0.035	[0.029,0.04]	0.0034
not_in_tune	0.05	[0.044,0.05]	0.018	[0.013,0.023]	0.0034
few_activity	0.045	[0.045,0.046]	0.026	[0.021,0.03]	0.0034
shop	0.04	[0.033,0.046]	0.086	[0.077,0.093]	1
alone	0.036	[0.033,0.036]	0.035	[0.029,0.04]	0.0034
map	0.034	[0.021,0.034]	0.018	[0.009,0.022]	0.0034
bath	0.029	[0.024,0.032]	0.071	[0.062,0.077]	1
not_common	0.029	[0.023,0.029]	0.045	[0.039,0.051]	1
not_close_partner	0.027	[0.024,0.031]	0.026	[0.02,0.035]	0.0034
no_children	0.026	[0.021,0.026]	0.011	[0.008,0.015]	0.0034
not_understood	0.025	[0.021,0.025]	0.035	[0.029,0.04]	1
low_con_child	0.022	[0.019,0.022]	0.011	[0.008,0.015]	0.0034
dress	0.02	[0.019,0.023]	0.046	[0.04,0.06]	1
cellphone	0.012	[0.011,0.013]	0.035	[0.029,0.04]	1
lack_companion	0.012	[0.01,0.012]	0.018	[0.014,0.022]	1
no_ppl_close	0.011	[0.008,0.011]	0.035	[0.03,0.04]	1
walk	0.011	[0.01,0.014]	0.045	[0.039,0.051]	1
low_con_fri	0.01	[0.01,0.013]	0.011	[0.008,0.015]	1
no_oth_fam	0.008	[0.008,0.008]	0.002	[0.001,0.005]	0.0034
meal	0.007	[0.006,0.008]	0.035	[0.029,0.048]	1
not_partnered	0.006	[0.006,0.006]	0.035	[0.029,0.04]	1
toilet	0.005	[0.004,0.005]	0.035	[0.029,0.04]	1
grip_low	0.002	[0,0.002]	0.002	[0,0.005]	1
eat	0.001	[0.001,0.001]	0.018	[0.014,0.022]	1
no_ppl_talk	0	[0,0]	0.018	[0.013,0.022]	1
left_out	0	[0,0]	0.006	[0.004,0.009]	1
no_friends	0	[0,0]	0	[0,0]	1
no_ppl_turn	0	[0,0]	0.011	[0.008,0.015]	1

S5-2 Table. 95% Confidence Interval of Strength from 10,000 Bootstrap Results

Node	Observed Network		Null Network (average)		p (observed > null)
	Bootstrap Mean	Bootstrap 95% CI	Bootstrap Mean	Bootstrap 95% CI	
shop	9.984	[9.881,10.057]	8.054	[7.34,8.613]	0.0034
not_part_group	8.058	[8.047,8.578]	8.059	[7.409,8.804]	1
medication	8.033	[7.994,8.158]	6.593	[5.846,7.086]	0.0034
dress	7.629	[7.521,7.893]	5.876	[5.328,6.837]	0.0034
bath	7.562	[7.538,7.584]	7.325	[6.726,7.846]	0.0034
not_understood	6.737	[6.685,6.751]	5.127	[4.543,5.562]	0.0034
bed	6.61	[6.588,6.7]	5.859	[5.25,6.336]	0.0034
no_ppl_turn	6.53	[6.454,6.596]	2.929	[2.485,3.278]	0.0034
meal	6.521	[6.456,6.901]	5.14	[4.59,6.132]	0.0034
isolated	6.453	[6.346,6.606]	8.788	[8.16,9.36]	1
walk	6.421	[6.402,6.481]	5.862	[5.281,6.33]	0.0034
not_common	5.982	[5.971,6.005]	5.858	[5.266,6.334]	0.0034
toilet	5.953	[5.887,5.984]	5.127	[4.615,5.564]	0.0034
cellphone	5.709	[5.648,5.824]	5.125	[4.511,5.6]	0.0034
no_ppl_close	5.675	[5.666,5.711]	5.127	[4.596,5.56]	0.0034
lack_companion	5.565	[5.555,5.588]	3.663	[3.177,4.054]	0.0034
money	5.395	[5.195,5.96]	6.161	[5.337,7.053]	1
alone	5.295	[5.285,5.345]	5.13	[4.615,5.583]	0.0034
eat	4.889	[4.843,4.99]	3.663	[3.164,4.031]	0.0034
no_ppl_talk	4.875	[4.795,4.94]	3.66	[3.212,4.045]	0.0034
left_out	3.789	[3.783,3.794]	2.195	[1.853,2.5]	0.0034
not_partnered	2.666	[2.661,2.773]	5.126	[4.605,5.563]	1
bal_fail	2.633	[2.589,2.901]	6.174	[5.356,7.069]	1
not_in_tune	2.616	[2.6,2.657]	3.662	[3.227,4.102]	1
map	2.514	[2.126,2.608]	3.66	[2.715,4.046]	1
not_close_partner	2.493	[2.482,2.557]	4.396	[3.935,5.147]	1
no_friends	2.289	[2.285,2.413]	0.731	[0.517,0.916]	0.0034
no_children	2.28	[2.267,2.349]	2.928	[2.492,3.282]	1
low_con_fam	2.175	[2.168,2.209]	5.129	[4.582,5.58]	1
low_con_child	2.066	[2.002,2.082]	2.931	[2.536,3.273]	1
low_con_fri	1.703	[1.699,1.705]	2.93	[2.55,3.282]	1
few_activity	1.359	[1.35,1.434]	4.394	[3.908,4.823]	1
no_oth_fam	1.112	[1.107,1.129]	1.464	[1.132,1.697]	1
grip_low	0.506	[0.33,0.509]	1.463	[0.577,1.718]	1
