# Supplementary material for: Exploring the links between social connection and physical functioning among older Adults: A network analysis
Source: PLoS One. 2026 Mar 23;21(3):e0342656. doi: 10.1371/journal.pone.0342656 (PMC13008092; doi:10.1371/journal.pone.0342656)
Supplement: S1 Table — (ZIP) [file pone.0342656.s001.zip › S6 Fig.pdf]

S6 Fig Sensitivity Analysis of Spearman Rank Correlation of Centrality (vs.gamma=0.25)

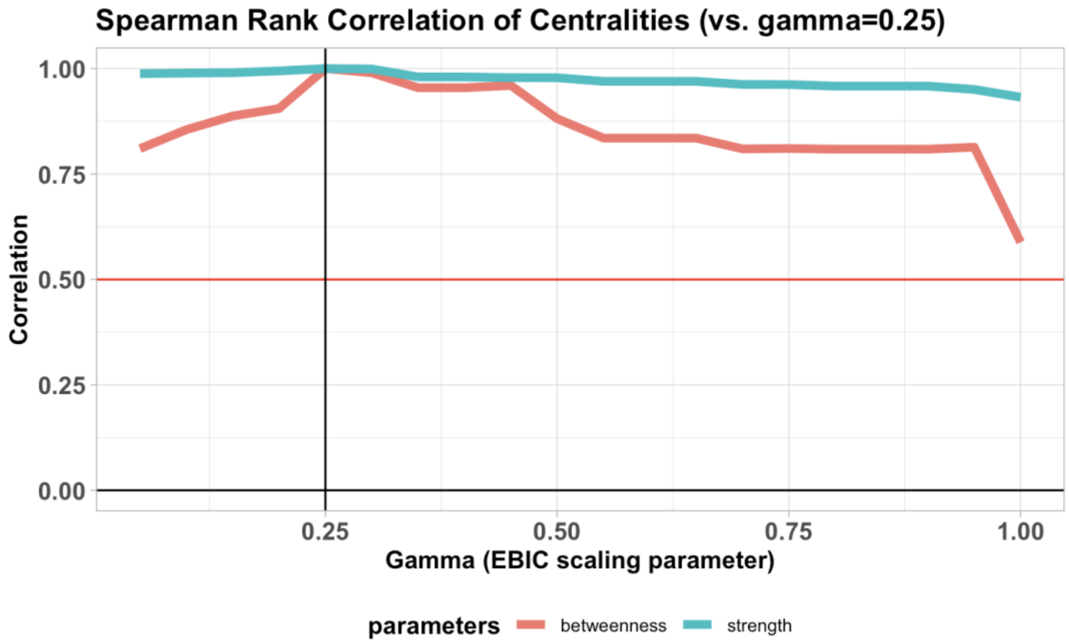

Note. On the X-Axis are different values of gamma from 0.05 to 1.00 with the interval of 0.05. The vertical black line (x=0.25) is the value of gamma used in the present study. On the Y-axis are the values of the Spearman Rank Correlation test between different values of gamma and gamma =0.25. The horizontal red line (y=0.5) is the threshold value of moderate correlation.
