# Supplementary material for: Exploring the links between social connection and physical functioning among older Adults: A network analysis
Source: PLoS One. 2026 Mar 23;21(3):e0342656. doi: 10.1371/journal.pone.0342656 (PMC13008092; doi:10.1371/journal.pone.0342656)
Supplement: S1 Table — (ZIP) [file pone.0342656.s001.zip › S7 Fig.pdf]

S7 Fig Plots of 95% Confidence Interval of Betweenness Centrality and Strength

S7-a Fig Connected Scatterplot of Betweenness Centrality among 34 Indicators (with 95% Confidence Interval)

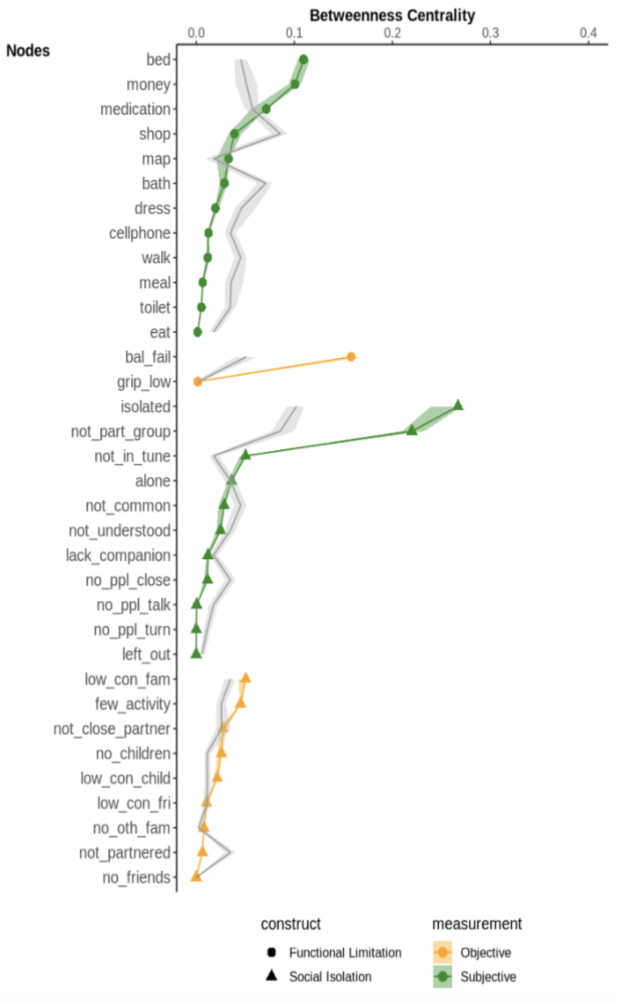

S7-b Fig Connected Scatterplot of Strength among 34 Indicators (with 95% Confidence Interval)

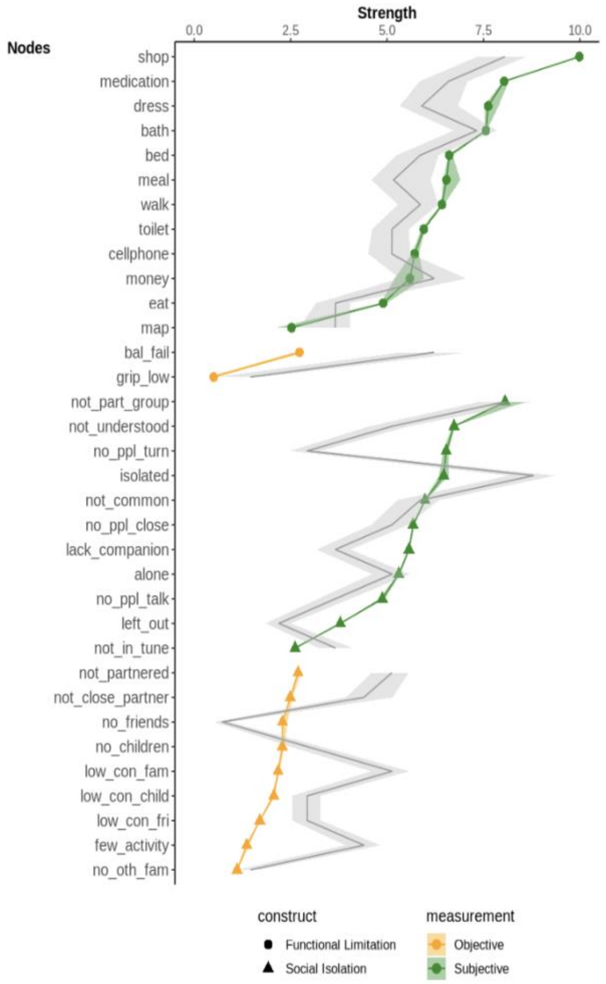

Caption for S7 Fig. Maize nodes refer to objective indicators (Io: Social connection/objective, Fo: Physical functioning/objective), and green nodes refer to subjective indicators (Is: Social connection /subjective, Fs: Physical functioning/subjective). Circles refer to physical functioning, and triangles refer to social connection. See **S2 Table** for node definitions and operationalizations. **S7-a Fig** showed the ranking of betweenness centrality values of 34 nodes, where nodes with the higher value were more important in linking the network. **S7-b Fig** showed the ranking of the strength value of 34 nodes, where nodes with higher value were more influential in the network. The gray line in the background is the mean of the parameter for each node estimated from 100 null models. The shade is the 95% bootstrap confidence interval of each estimate.
