# Supplementary material for: Exploring the links between social connection and physical functioning among older Adults: A network analysis
Source: PLoS One. 2026 Mar 23;21(3):e0342656. doi: 10.1371/journal.pone.0342656 (PMC13008092; doi:10.1371/journal.pone.0342656)
Supplement: Attachment — Submitted filename: Response to Reviewers.docx [file pone.0342656.s003.docx]

Reviewer #1: The researchers claim to have developed a new approach, namely to measure the relationships between different concepts with the help of network analysis. I found it interesting to become acquainted with this approach. My opinion is that the network analysis can be a valuable addition to existing possibilities, but that this paper still has too many flaws to be considered for publication at this time. (Theoretically, I am familiar with network concepts, but my expertise with applied network analysis is limited.)

Comments and suggestions

1. The article focuses on the link between social connection and functioning. However, functioning can be in all kinds of areas (including social); so it must be about physical functioning. Even then, the two terms are not completely valid. Loneliness is much more than social functioning, and also involves emotional functioning, for example. ADL and IADL are also more than just physical functioning, as is rightly argued in the article. The title of the article is therefore unclear and invalid.

Response: Thank you for your comment. We agree that the term “functioning” is unclear for readers. We meant to say physical functioning. In response, we have changed all mentions of “functioning” or “functional limitation” to “physical functioning." We have also revised the title to now be: Exploring the Links Between Social Connection and Physical Functioning Among Older Adults: A Network Analysis.

2. The introduction discusses the necessity of applying a network analysis. However, this is not convincing. For example, “empirical understanding of the linkages between these constructs is hampered by differences between ‘objective’ (e.g., grip strength, network size) and ‘subjective’ (e.g., activities of daily living, loneliness) measurement schemes”. What is the objection here? Isn't it strange that the applied network analysis leads equally well to a distinction parallel to objective and subjective concepts?

The second aspect, namely that other research ignores the multi-dimensional structure of concepts, is not convincing either. Contrary to what is stated on page 7, there is in fact a lot of research in which constructs in the two domains of functioning are multidimensional. It is noteworthy that the researchers themselves use the UCLA scale, which was developed as a unidimensional scale, while there are also various multidimensional loneliness instruments. The researchers also object to summary scores, but they use them themselves (Table S2).

Response: We agree with the reviewer that the Introduction did not deliver a convincing argument of why we applied network analysis. The intention of the argument in the introduction was neither to criticize different types of measurements nor to criticize that prior research relied on regression. Rather, our goal was to emphasize that network analysis provides a unique way to visualize item-level connections across constructs, in addition to considering constructs at the summed score level. This approach adds valuable information to understand the complex relationship between social connection and physical functioning.

Regarding reporting the summary score of the UCLA loneliness scale in the supplementary material, we anticipated that readers may also be interested in the overall scale, given its widespread use in prior literature.

To address this comment, we have substantially revised the first paragraph (lines 50 to 54) and the last two paragraphs of the Introduction (lines 88 to 121) to clarify our motivation.

3. It is not clear to me why network analysis is absolutely necessary, and why a confirmatory factor model has not been developed in which latent concepts are measured by different indicators, with relationships between the latent concepts.

Response: We did not mean to imply that network analysis is the *only* analytic approach that could address this question; rather, we only propose that it is an *appropriate* one to use for our research question. In addition, we feel that confirmatory factor analysis (CFA) and psychometric network analysis answer related, but distinct, research questions. Specifically, CFA requires the assumption of an underlying latent concept, which is best-suited for research questions that have a strong hypothesis-testing goal. By contrast, psychometric network analysis does not assume the latent concepts; rather, it is a more exploratory tool to visualize the associations among individual indicators. This is more reflective of the goals of our analysis.

In response to this comment, we have now added a sentence to the Discussions section acknowledging that other tools (like CFA) are also relevant for understanding the inter-relationships between social connection and physical functioning in lines 399 to 404.

4. On page 6, the researchers again express objections to an existing technique, this time to regression-based approaches. Unfortunately, they do not explain why these are unsuitable or why they do not lead to improved interventions. The researchers should better substantiate their criticism of other methods and not selectively cherry-pick from those other methods. It would also be preferable to discuss this in one section rather than scattered throughout the paper.

Response: We appreciate the opportunity to clarify our intent. The goal of this section on page 6 was to emphasize the advantages of network analysis as a tool for visualizing and analyzing item-level connections across constructs, which regression is not well-suited for. However, we acknowledge that this intent was not clearly conveyed in the initial text.

Therefore, in response to this comment, we have substantially revised the Introduction (including said text regarding regression) to emphasize our motivation in a more balanced manner. Please see lines 88 to 121.

5. The researchers see their measurements of ADL and IADL functioning as subjective. I do not share this opinion. This is about objective functioning, as assessed by survey questions. I am familiar with another study using a more extensive set of performance measurements and including observations of a number of ADL and IADL functions. This indicates that the phenomenon itself can be observed objectively – of course, bias can arise when questions are asked in a survey.

Response: We agree that ADL and IADL can be objectively assessed (e.g., through direct performance tests or observation). In the HRS, however, the ADL and IADL were only assessed as *self-reported questions (i.e.,* “Because of a health or memory problem, do you have any difficulty with getting in or out of bed?” with response options including *yes, no, can’t do,* and *don’t do).* This is what we intend to capture by referring to these ADL/IADL measures as “subjective,” because this way of measuring them is distinct from the “objective” measures which, in the HRS, were measured via *standardized physical performance tests* (e.g., time standing as part of a balance test, amount of force exerted during a direct test of grip strength).

We agree that our intent in using the terms subjective/objective for the various measures of physical functioning needs to be clearer. In response to this comment, we have revised the method section at lines 148-155.

6. I also find it unbalanced to use two performance measurements and twelve survey questions. The fact that twelve questions are needed to measure ADL and IADL functioning already indicates that the content of these concepts is much broader than being able to balance and grip strength. This requires a much more precise conceptualization of physical functioning.

Response: We agree that the imbalance between the number of subjective and objective indicators raises some concerns, and further, we acknowledge that twelve subjective indicators encompass a much broader range of physical functioning than the two objective indicators. Our goal was to take advantage of all the relevant physical functioning measures (both self-report and performance-based) in this dataset. Unfortunately, the HRS dataset only included two measures of physical performance (balance test and grip strength).

We agree that additional physical performance tests in the network would have yielded more nuanced insights. Therefore, in response to the comment, we have added a sentence in the Limitations section to acknowledge the issue and noted that future studies should include a more balanced set of indicators in lines 423 to 427.

7. The researchers distinguish two components of social connection, namely structural characteristics and perceived quality. However, the literature is more accurate than what is currently presented. There are (at least) structural and functional (e.g. social support and care) characteristics, and the perceived quality of both can be determined. These are different theoretical concepts (or constructs – that is a synonym), and it is incorrect to interpret the differences as merely a difference in measurement (page 6, line 125).

Response: We acknowledge that the literature on social connection has been updated since our manuscript was submitted and reviewed. We agree with the reviewer that social isolation and loneliness are not different measures of the same construct, but different measures of sub-concepts of social connection (structure and function).

In response, we have substantially revised the relevant text and updated the citations in lines 75 to 85.

8. The selection in Figure S1 is very crudely displayed. What is the consequence of the selective dropout?

Response: We agree that Figure S1 can benefit from adding more detailed information. We have updated Figure S1 to provide a clearer description of participant exclusions.

Specifically, the sample excluded 19,872 participants because the “left-behind” questionnaire was administered to only a random half of the participants. We excluded 6,365 participants with missing values on any study variable because the network analysis required complete data.

As a result, the analytic sample was more likely to have higher SES, to be younger, and to be non-Hispanic White. Thus, our estimates may be subject to selection bias, and the findings may have limited generalizability. In response, we have added sentences in the Limitation in lines 427 and 430.

9. Why have all indicators been dichotomized (page 8)? If this is necessary for the technique, does not the removal of much variance then present an objection to the choice of network analysis?

Response: We acknowledge that dichotomization is not required for network analysis. However, for our analysis, we decided to use dichotomization based on the distribution of the majority of variables and statistical considerations:

First, several indicators were already binary or near-binary in their *original scale*. For example, the original options for the ADL and iADL are binary (Do you have any difficulties in [name of activity]? *Yes* vs. *No/Can’t do/Don’t do*). Also, the original options for several social connectedness questions are also binary (e.g., Do you have friends? *Yes* vs. *No*. Do you have children? *Yes* vs. *No*).

Second, for many other measures, although the original scale was continuous, the distribution was heavily skewed. For example, ~80% of values on the balance test (which is recorded continuously as time in seconds) were at their age-specific maximum time: 40 seconds or 70 seconds.

Third, although network analysis allows mixed types of variables to be the nodes, in our attempts to do this, we had issues with model convergence.

Finally, although using all-binary variables to indicate the nodes reduces within-variable variance, our analytic approach is primarily interested in *between-variable covariance*. This includes between the constructs of social connectedness and physical functioning more broadly (e.g., identification of bridging nodes), but also within these two constructions (e.g., identification of communities/subgroups within the constructions).

We addressed this comment by revising the Discussion to acknowledge the limitations of our approach in lines 422 to 423.

10. The UCLA scale has twenty items, not eleven (page 9). Why this selection, and on what grounds were these eleven items selected?

Response: We acknowledge that the original UCLA scale has 20 items. However, the HRS used an 11-item version of this measure, which derived 11 items from the full UCLA scale, as described here:

Smith, J., Ryan, L., Fisher, G., Sonnega, A., Weir, D., Weir, D., & Weir, D. (2017). *HRS Psychosocial and Lifestyle Questionnaire 2006-2016*. Survey Research Center, Institute for Social Research, University of Michigan.

This citation also reported that the 11-item version of this scale has a high Cronbach’s alpha of 0.87.

We apologize that we only cited the 20-item version in the initial version of the paper. In response to this comment, we have now added the Smith (2017) citation to the manuscript to make our methods more transparent.

11. What is the reliability and validity of the Social Disconnectedness Scale?

Response: According to Cornwell and Waite, the reliability of validation of the social disconnected scale is 0.73 (cite). In response, we have added Cronbach’s alpha to the manuscript in line 176.

Cornwell, E. Y., & Waite, L. J. (2009). Measuring Social Isolation Among Older Adults Using Multiple Indicators From the NSHAP Study. The Journals of Gerontology Series B: Psychological Sciences and Social Sciences, 64B(Suppl 1), i38–i46. <https://doi.org/10.1093/geronb/gbp037>

12. What technique was used to adjust for demographic covariates in the relationships between the 34 indicators? Why was the measurement level of these covariates lowered?

Response: We used the glmet () function to generate the network, and we used the argument “penalty.factor” to adjust for the demographic covariates. We adjusted for demographic covariates because we want to adjust for potential confounders that could influence the estimates of edge weights.

We categorized continuous variables (e.g., wealth) into bins to avoid assuming a linear relationship between those variables and the node.

13. If less than 10% of respondents had any ADL or IADL limitations, is this sufficient to be able to carry out the analyses?

Response: We understand the reviewer’s concern about power. However, all of the models converged, including those in the sensitivity analysis, which suggests that the sample was sufficient to carry out the analyses.

14. The representation in a two-dimensional space was done with the spin glass community detection algorithm – it is unclear to me what the length of the lines means and to what extent figure 2 differs from figure 1. The display reminded me of the placement of items or other units in a two-dimensional space as done in an old program called MINISSA (https://www.newmdsx.com/MINISSA/minissa.htm) based on Euclidean distance. To what extent do the results of the network analysis differ from such a graphical representation?

We agree that the explanation of the spin glass algorithm, used to detect communities (represented by colors in Figure 2) but not the layout, can be clearer. The layout of the network was generated using the Fruchterman–Reingold layout, the goal of which is to create a balanced visualization of the network(1). The length of the lines could possibly change if we change to a new seed number.

The difference between Figure 2 and Figure 1 is the color of the nodes, not the length of the lines. In Figure 1, the color of the nodes was pre-determined by constructs (physical functioning / social connection), so there were two colors. In Figure 2, the number of colors and the colors of each node were determined by the spin glass community detection algorithm. The algorithm assigns the same color to the nodes that have stronger correlations with each other.

The value of having community detection is that it adds more information about how nodes were connected together, showing us that nodes from the same scale aren’t interchangeable, and there might be a subgroup.

In response, we have added additional information about the community detection algorithm in Method, in lines 221 to 225, and clarified the interpretation in the Results section, in lines 297 to 308.

Reference: <https://karobben.github.io/2023/02/20/LearnNotes/fruchterman-reingold/>

15. The accessibility of the results would benefit from a clear explanation of what they mean in terms of content.

Response: We agree that the manuscript would benefit from a clearer explanation of the results in context. Therefore, we made significant revisions in the following sections: 1) Introduction: We have now clarified the motivation and research aim of the study (lines 87 to 121). 2) Methods: We have reduced technical terms and added concrete examples to illustrate quantitative measures, such as the assortativity index (lines 214 to 220). 3) Results: We have added descriptive sentences to guide readers through the data visualizations, and contextualized the quantitative network measures in the relationship between social connection and physical functioning (lines 273 to 307). 4) Discussion: We have expanded the text describing how our findings contribute to the field and potential practical implications(lines 359 to 396). Please see the revised manuscript.

16. The conclusion is drawn that “that falling the balance test played the most important role in linking functioning and social connection” (page 18). (Should it be "failing"?) But looking at figures 1 and 2, I do not see that. On what is this conclusion based, and why is this not visible in the figures?

We agree that our conclusion about the balance test could be clearer. The conclusion that the balance test played the most important role in linking physical functioning and social connection, drawing from our finding that the node balance test has a high betweenness centrality. Along with the ablation test, removing the node of the failing balance test significantly increased assortativity, meaning that without failing the balance test, the two constructs, physical function and social isolation, would be significantly less connected.

In response, we have revised the Result section in lines 325 to 330 to better explain the results and the Discussion section in lines 356 to 358 to conclude more clearly.

17. What does the conclusion “Collectively, these findings suggest that functioning and social connection have a multifaceted association that reflects both performance-based functioning and perceived aspect of social connection” add to existing knowledge in this domain?

Response: We appreciate the opportunity to clarify the contribution. Our analysis identified that the balance test is the key bridge item between social connection and physical functioning, rather than grip strength or other task-oriented physical functioning measures (e.g., ADL/ iADL). Given that much of the existing work relied on aggregated scores, this conclusion pinpointed a specific performance-based measure. The implication is that improving balance capacity is relevant not only for interventions on physical functioning but also for those targeting social connection.

In response, we have expanded our discussion about the implications of our findings to the Discussion section in lines 375 to 396.

18. Why is it possible to use this study based on data from older adults to make suggestions for interventions for “struggling students in public elementary schools”?

Response: We agree that our statement about the “CORP” intervention can be clearer. The Experience Corps® is a community-based program designed for older adults, not elementary school students. In this program, participants (older adults) are first trained in exercising, social, and cognitive aspects with peers, and then volunteer as tutors in the local public elementary school to help struggling students. We mentioned the program because it is a strong example of an intervention that targets both physical functioning and social connection in older adults. Furthermore, the program did show significant improvement in participants' mobility and social engagement.

In response, we have added additional information about the Experience Corps® program in the Discussion in lines 386 to 396.

19. Quality of writing. There are many errors in spelling or in sentence structure. This indicates sloppiness in dealing with texts and possibly also in dealing with data.

We apologize for the weaknesses in the writing of the initial submission of this paper. We have substantially revised the text throughout, as noted in our above responses. Thank you for your patience.

20. References. This needs to be improved. I will give a few examples. A sentence such as “For example, older adults with functional limitations are more likely to experience shrinkage of life-space and decreased social activities.” needs elaboration and more references. No reference is given for the definition of loneliness (it says: “(reference)”).

Thank you for pointing these out. We have added the following paper as the reference.

Chum, A., Teo, C., & Azra, K. K. (2022). Does the longitudinal association between neighbourhood cohesion and mental health differ by ethnicity? Results from the UK Household Longitudinal Survey. Social Psychiatry and Psychiatric Epidemiology, 57(4), 859–872. <https://doi.org/10.1007/s00127-021-02125-6>

21. On page 4, lines 88 and 89, sources are mentioned, but these are not good references.

We agree that the references cited in lines 88, and 89 were not the most appropriate. We have replaced them with more relevant and recent sources that directly support the statement.

22. On what do the authors base the conclusion that “the precise mechanisms underlying these relationships are unclear” (page 4)? The association between social connection and health outcomes has been extensively researched, and fortunately much has also been done to work out the underlying theoretical mechanism. On page 5 it is stated: “the empirical research on this relationship is mixed” (I think this must refer to the results of the research).

Response: We agree that there is a substantial theoretical literature on pathways linking social connection to health, such as chronic stress or sleep disruption. Our intent was not to imply an absence of research, but to note that the causal relationship between the two constructs, as well as potential modifiers and mediators, has not been fully established. We also acknowledge the complexity of this association between social connection and physical functioning, given that both constructs have multiple layers.

In response, we have revised the Introduction to state this more precisely and added representative citations in lines 88-110.

23. Not only is this statement contrary to the conclusion that the relationship is unclear, but it is also not true that the results are mixed. In the example mentioned, in which ELSA and HRS are compared, it concerns two very different outcomes, probably with different underlying mechanisms as well.

Response: We acknowledge that it is not appropriate to cite the HRS study that investigated cognitive decline. In response, we have replaced it with another study using HRS that investigated the relationship between loneliness, social isolation, and some physical outcomes.

24. The description of the Disablement Process Model mentions “intra-individual factors (e.g., loneliness)”, but loneliness is not mentioned in either of the two referenced publications.

We agree with the reviewer’s concern about the reference. The original disablement process model was published in 1993, when loneliness was not a widely discussed concept. We believe that loneliness belongs to “intro-individual factors” because it is a perceived feeling, and belongs to one’s psychological status. In response, we have added a reference paper that also investigated whether loneliness mediates the physical functioning decline based on the disablement process model.

Reference paper: Fauth, E. B., Zarit, S. H., & Malmberg, B. (2008). Mediating relationships within the Disablement Process model: A cross-sectional study of the oldest-old. European Journal of Ageing, 5(3), 161. <https://doi.org/10.1007/s10433-008-0092-6>

25. Ethics of the research. I see no reference to a publicly available report on the processing of the original data.

Response: We only share the link to the dataset at the submission portal. We agree that providing the link in the manuscript would be helpful. In response, we have added the link in line 139.

Reviewer #2:

1. Novelty:

1. The study presents an innovative approach by examining both subjective and objective indicators of social connection and functioning using network analysis, moving beyond traditional regression methods.

The identification of balance impairment and loneliness as key bridging nodes between functioning and social connection provides valuable, actionable targets for intervention and policy development.

The manuscript would benefit from a more in-depth exploration of the potential mechanisms underlying the strong association between isolation and imbalance.

Response: We agree that a more in-depth exploration of the potential mechanism between isolation and the balancing problem is needed. In response, we have added sentences to illustrate a potential mechanism in the Discussion in lines 375 to 385.

2. Further elaboration on the real-world applicability of identifying the bride node and their capacity to inform targeted intervention strategies is advised. Explicating how these findings can be translated into concrete, actionable recommendations would greatly enhance the paper’s value for both practitioners and policymakers.

Response: We agree with the reviewer that the discussion will be beneficial from elaboration on the potential real-world application of the bridge and how the findings can translate into recommendations.

In response, we substantially expanded our Discussion in lines 386 to 396.

3. Conceptual Framework & Operationalization:

The study is well-grounded in a sound theoretical framework linking physiological impairments with the social environment, and the research question is clearly situated within existing literature. The distinction and precise operationalization of objective versus subjective indicators strengthen the analytical rigor and provide nuanced insight. However, the framework would benefit from clarification on the rationale for classifying perceived loneliness as an objective indicator, as this appears counterintuitive.

Response: Thank you for the opportunity to clarify the classification of loneliness. In the study, we assessed loneliness using the UCLA scale, which is an 11-item self-reported questionnaire. Therefore, we classified this as a subjective indicator. We recognize that we need more clarity on how we classified nodes. In response, we have revised the text to clarify how we classify types of measurements.

4. Generalizability

The exclusion of participants who were more socioeconomically disadvantaged and from minority groups may introduce bias into the study. It may be helpful for the authors to explain the rationales, reflect on the potential impact of this exclusion on their findings, or recognize it among the study’s limitations.

Response: We agree that the analytic sample was more likely to have higher SES, be younger, and be white. In response, we added the additional sentences in the Limitations in lines 427 to 430 to acknowledge this and its implications for the generalizability of our results.

5. Measurement Dichotomization:

The dichotomization of continuous or ordinal variables (e.g., grip strength) may reduce variability and sensitivity, potentially limiting the precision of the analysis. The authors are encouraged to provide a rationale for this approach or consider alternative analyses utilizing raw or more finely graded data.

Response: Please refer to comment #9 from Reviewer 1 above and corresponding revisions in line 422 to 423.

6. Measurement Selection

To strengthen the methodological rigor, it would be beneficial for the authors to provide a justification for the selection of these specific measures by referencing their theoretical relevance or reporting on established psychometric properties (e.g., reliability or validity from previous empirical studies). Providing such rationales would enhance the interpretability of the findings.

Response: We agree that adding psychometric properties will be beneficial. We have added Cronbach’s alphas for both subjective and objective measures of social connection (line 168, 176), subjective measures of physical functioning (line 154); we have also added intra-rater reliability for two physical performance measures (line 164) to the manuscript in the Method section.

7.Rigor in Analysis:

The application of appropriate network estimation techniques, comprehensive stability assessments, and adjustment for relevant covariates collectively strengthens the validity and reliability of the findings. Although sensitivity analyses for γ were conducted, a clearer rationale for choosing the default value of 0.25 and an interpretation of what increasing or decreasing γ entails for network sparsity would strengthen the methodological transparency.

Response: We agree that a clearer explanation of how the network change corresponds to the change of γ will be beneficial. In response, we added a sentence to the Method on lines 206 to 208.

8.Community Interpretation:

The manuscript’s impact could be enhanced by offering a more in-depth interpretation of the identified communities, including potential underlying mechanisms, theoretical implications, and alignment with prior research. Additionally, discussing the relevance of these communities for designing targeted interventions would add practical significance.

Response: We agree that the result of community detection needs more in-depth interpretation. In response, we have expanded interpretations of community detection, theory, and potential implications on intervention in the Discussion in lines 359 to 374.

9. Proofreading

You are suggested to proofread the word in detail (e.g., spelling, grammar, spacing, the statistic in tables, etc)

Response: We agree that there are several typos; we fixed them this time and conducted a thorough proofread in this round of revision.
