# Supplementary material for: Exploring the links between social connection and physical functioning among older Adults: A network analysis
Source: PLoS One. 2026 Mar 23;21(3):e0342656. doi: 10.1371/journal.pone.0342656 (PMC13008092; doi:10.1371/journal.pone.0342656)
Supplement: Attachment — Submitted filename: RR2_Response to Reviewer.docx [file pone.0342656.s004.docx]

Reviewer #2: Review Comments

1. • The study demonstrates methodological rigor by employing network analysis alongside additional analyses (e.g., bootstrapping, ablation testing, null model comparison), enhancing confidence in the robustness of results.
2. • Multiple measurement modalities (objective and subjective metrics for both constructs) are presented, allowing nuanced assessment and triangulation of results.
3. • The use of network analysis in this domain is innovative and provides added value by identifying bridging indicators that inform targeted, multi-modal interventions for older adults.

Response: We thank the reviewer for their positive and encouraging comments!

4. The manuscript’s conceptualization of “subjective” measurement is not fully resolved. It would enhance clarity to directly discuss whether perceived difficulty is equivalent to subjectivity and to distinguish environmental influence (e.g., adaptive home environments reducing perceived difficulties) from inherently subjective self-perception. This distinction is important for interpreting responses to ADL/IADL questions and understanding measurement validity.

Response: We appreciate the opportunity to clarify our conceptualization of the subjective measure of physical functioning. Our “subjective” measures of physical functioning include several ADL/IADL questions. These questions are self-reported, and respondents judge their level of difficulty in performing a specific activity of daily living on an original 4-category scale: No, Yes, Can’t do, Don’t do.

Beyond subjectivity (i.e., self-reported), we consider these ADL/IADL measures subjective because they reflect respondents’ actual physical abilities as well as their perceptions and evaluations of environmental constraints or adaptations. While the subjective measures in our study did not distinguish between environmental influences and self-perception, we believe that our conceptualization of subjective measures reflects how individuals evaluate their lived experience of physical functioning difficulties in real-world settings.

However, we agree that we need additional clarity on our conceptualization of subjective measurement. In response to this comment, we have revised the conceptual definition of subjective measurement on page 3 (lines 64-69) to more clearly reflect our perspective.

5. The rationale for choosing psychometric network analysis over conventional regression-based approaches remains insufficiently developed. It is recommended to explicitly state what methodological gaps or limitations in prior literature necessitate network analysis for these constructs, beyond precedent in mental health symptom comorbidity research.

Response: We agree with the reviewer that the introduction section would benefit from a more robust justification for our choice of network analysis over conventional regression-based approaches. In previous revisions, we aimed to strike a balance that highlighted the novelty of our approach without overlooking the foundational contributions of traditional regression-based research.

To integrate this recommendation, we have added a paragraph on page 5 (lines 113 to 125) to explicitly state the methodological gaps in the literature and clarify how network analysis provides complementary insights rather than replacing conventional regression-based approaches.

6. Clarify the unique insights network analysis can provide regarding the multidimensional relations between physical functioning and social connection, such as detecting communities, examining individual indicator centrality, or visualizing complex patterns that are not accessible using aggregated regression models.

Response: We agree with the reviewer that the unique insights offered by network analysis will strengthen the manuscript. We have addressed the comment throughout the paper:

1) **Introduction:** We have added a sentence about how network analysis addresses methodological limitations in conventional regression-based models on page 5 (lines 113 to 124).

2) **Method:** We have added justification for each of the three metrics, highlighting their potential theoretical and practical implications on page 10 (lines 229 to 247).

3) **Discussion:** We have added several sentences to clarify the unique insights provided by our network analysis that would not be accessible to other traditional models on page 16 (lines 369 to 371), page 17 (lines 387 to 390), and page 17 (lines 399 to 400) .

7. The statement that excluded participants were more likely female, racial/ethnic minorities, or of lower socioeconomic status warrants further elaboration. It is advised to provide a transparent rationale for these exclusions, as it has implications for the representativeness and generalizability of the findings.

Response: We agree that a more detailed statement of excluded participants is necessary for transparency. Given that information on 22/34 nodes was derived from a self-administered questionnaire and the network analysis only allows complete cases, a significant portion of respondents was excluded. To address the comment, we have expanded the sentences of our analytic decision to the Method section on page 7 (lines 148 to 149). We also discussed the potential limitation of the exclusion criteria in the Limitation section on page 19 (lines 446 to 449).

8. • While the manuscript defines network indices (strength and betweenness), it insufficiently addresses their selection and utility. You may consider justify why these specific indices were chosen and discuss how findings from strength vs. betweenness analyses advance theoretical or practical understanding in this context.

Response: We agree with the reviewer that a more detailed justification of our choice of network metrics (the strength and betweenness) provides additional clarity for the readers. We have added sentences to clarify our choice and have reorganized the entire paragraph to provide clearer motivation for each of the three network features on page 10 (lines 229 to 247). Our response to comment #6 partially addresses this comment as well.

9. • Greater attention is needed to grammatical accuracy and tense usage, particularly in sections describing data collection (should use the past tense).

Response: We thank the reviewer for pointing this out. We have revised the "Sample" sections to ensure consistent and correct use of the past tense on page 6 (lines 139 to 145). Additionally, we have performed a thorough line-by-line grammatical review of the entire manuscript to ensure accuracy and professional tone.
